# Cerebral Hemangiopericytoma Manifesting as Epilepsia Partialis Continua: A Case Report

**DOI:** 10.31729/jnma.7410

**Published:** 2022-06-30

**Authors:** Prabhat Poudel, Sushan Shrestha, Maya Bhattachan

**Affiliations:** 1 Nepal Medicai College and Teaching Hospital, Attarkhel, Kathmandu, Nepal; 2 Department of Neurosurgery, Kathmandu Medical College and Teaching Hospital, Sinamangal, Kathmandu, Nepal; 3 Department of Neurosurgery, Nepal Medical College and Teaching Hospital, Attarkhel, Kathmandu, Nepal

**Keywords:** *case reports*, *epilepsia partialis continua*, *hemangiopericytoma*, *solitary fibrous tumors*

## Abstract

Cerebral hemangiopericytomas are very rare mesenchymal tumours arising from pericytes surrounding the blood vessels in the brain. Most patients present with headaches, focal neurological findings and focal seizures with or without generalisation. Our patient chiefly complained of an uncontrollable movement of her right hand that was initially fleeting but later became continuous. Her symptoms were initially described as tremors. We found an intracranial tumour as a cause of her symptoms, suspected the tumour to be a meningioma and performed surgical extirpation which resulted in symptom resolution. Histopathology and immunohistochemistry of the excised mass revealed that the tumour was hemangiopericytoma. The patient is being closely monitored for recurrences and metastasis. Hemangiopericytomas are very rare and they rarely result in the abnormal movements of epilepsia partialis continua. Differentiation of the abnormal movements of epilepsia partialis continua from tremors is very important as is the differentiation of the tumour from meningioma.

## INTRODUCTION

Cerebral Hemangiopericytomas (HPCs) are very rare mesenchymal neoplasms arising from Zimmermann pericytes surrounding blood vessels.^1^ They comprise 0.4% of Central Nervous System (CNS) tumours.^2^ Intracranial HPCs are commonly supratentorial, virtually indistinguishable from meningiomas by imaging alone, and were initially considered as angioblastic variants of meningioma. Due to the rarity of this neoplasm, the natural history, management and follow up guidelines remain mostly undefined.^3^ Epilepsia Partialis Continua (EPC) is a rare cortical seizure, best described as a status epilepticus equivalent of a focal motor seizure with retained awareness.^4^ Here, we present a case of a lady who presented with EPC as a manifestation of cerebral hemangiopericytoma.

## CASE REPORT

Our patient is a 34-year-old lady who complained of having abnormal uncontrollable movement of her right hand for 12 days. Her symptoms were acute on the onset, limited to the right wrist joint and the joints of the right hand, including distal to the wrist joint. Initially, the movements would last for a few seconds to a few minutes, followed by weakness of the involved hand and forearm muscles. A few days after the onset of her symptoms, the movement changed from an on and off type to a rather continuous type, being present even during her sleep. The movements were unorganised, had no fixed axis and did not show any particular rhythm, had no relation to the position or use of her right forelimb, never involved other proximal joints of the right forearm and never generalised into a full-blown seizure. She has always been aware of the movements but was never able to voluntarily suppress them. We came to the conclusion that her movements were like that of epilepsia partialis continua.

On further questioning, she revealed that she has had mild dull headaches in the bifrontal region for a long time, although she has never been too bothered by the headache and has been taking over-the-counter analgesic medications for pain control. She gave no history of having fever, feeling nauseated, vomiting, developing a prodrome before headache episodes, or losing her consciousness. No other significant findings on general and systemic examination. Her vitals were stable. There was no significant family history.

**Figure 1:**
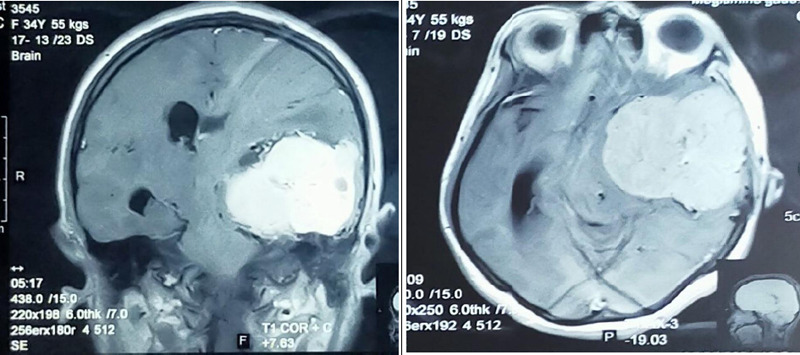
Contrast-enhanced MRI of the brain showing the left-sided hemangiopericytoma with mass effects.

Magnetic Resonance Imaging (MRI) of the brain revealed a large heterogeneous lobulated extra-axial mass involving the left parietal and temporal region with a midline shift of 9 mm to the opposite side, skull vault erosion, brain stem rotation and white matter oedema (Figure 1).

The mass had thickened and enhanced the dura adjacent to it. On Computed Tomography (CT) correlation, the mass appeared relatively hyperdense with a focus on calcification within and it appeared to have caused the erosion of the greater wing of the sphenoid bone and squamous part of the left temporal bone. After our initial evaluation of the images, the mass was expected to be a meningioma, but we could not be sure until surgical extraction of the mass and histopathological examination were performed.

She underwent craniectomy with gross total tumour excision and had a good recovery postoperatively. Intraoperatively, the lesion was highly vascular with clear margins and the mass adherent to the tentorium cerebelli. She had a good post-surgical recovery. The abnormal movements of her hand were no longer present and she was discharged after she showed significant improvement. She is now on a regular follow up and is being closely monitored for recurrence or metastasis of the tumour, fortunately, none of which has been found to date.

**Figure 2:**
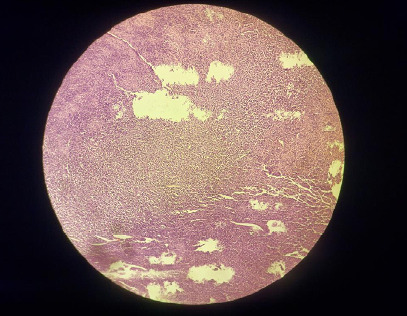
Histopathological slide of the tumour demonstrating sheets, bundles, fascicles, nests and alveolar pattern of tumour cells with ectatic vessels traversing the tumour.

Histopathological examination of the excised mass demonstrated diffuse sheets, bundles, fascicles and nests of ovoid to elongated tumour cells in whorls and a vague storiform pattern with vesicular chromatin and also an alveolar pattern of cells (Figure 2).

Numerous ectatic thin-walled blood vessels were found traversing the tumour, suggestive of hemangiopericytoma. The diagnosis was confirmed with immunohistochemistry that was positive for CD34, vimentin and CD99; negative for EMA and HMB45; and Ki67 index was less than 1%. Recurrence and metastasis are being monitored at regular follow-up visits. On the last follow up, we had a brief discussion with the patient about her perspective on the care she received from us. She was very pleased by the control of her symptoms and expressed very warm gratitude towards the people involved in her care.

## DISCUSSION

Cerebral Hemangiopericytomas are rare aggressive mesenchymal neoplasms and the mechanisms underlying the development of hemangiopericytoma are poorly understood.^1,5^ Their clinicopathological characteristics and prognosis are mostly unknown and the guidelines for diagnosis-management and follow up remain unestablished.^6,7^

The mean age for diagnosing HPC is about 44 years; in our case report the age was 34 years.^3^ In most of the literature published on cerebral HPC-headache is the most commonest symptom-however our patient did not give any history of headache on her own but when asked about it during a clinical interview-she revealed having on and off mild headaches throughout the last year-much similar to the headaches she would occasionally have in earlier years-but was never too bothered by them so never sought any medical care until she developed those abnormal movements of her right hand. After a thorough review of the literature, we were unable to find any publication with EPC as the sole presentation of intracranial hemangiopericytoma.

Common differential diagnoses for cerebral HPCs are meningiomas and other variants of solitary fibrous tumours. The clinical behaviours of HPCs are more aggressive than that of benign meningiomas and have a strong tendency to local recurrence and extracranial metastasis; therefore it is vital to distinguish between these two.

On brain CT, cerebral hemangiopericytomas appear as hyperdense lesions with lobulated margins and broad base meningeal attachment, very similar to and difficult to distinguish from meningioma.^8,9^ However, intracranial meningeal hemangiopericytomas are more multi-lobulated than benign meningiomas and have thinner based dural attachment on CT-scan and MR images.^10^ Normally, they have intratumoral vessels and have a strong mass effect.^11^

Histopathologically, HPCs show a staghorn pattern of spindle cells^11^ and immunohistochemical staining shows strong positivity with vimentin (100% of cases), CD99 (94% of cases), and CD34 (88% of cases) but only focal positivity with Epithelial Membrane Antigen( EMA) (33% of cases).^12,13^ Meningioma however is strongly positive for both vimentin and EMA. STAT6 immunohistochemistry with sensitivity (96% ) and specificity (100%) is a reliable diagnostic marker of HPCs.^14^

Surgical resection of intracranial HPC, in an attempt to reach Simpson grade 1 removal of the tumour, is necessary to reduce recurrence and adjuvant radiation may show promise in preventing tumour progression.^15^

The risk of local tumour recurrence is significantly higher than neural axis metastases of the CNS and extraneural metastases; however, the risk of distant delayed metastases can reach up to 23%,^3^ so it is very important to have a strict follow-up schedule for patients with diagnosed with this neoplasm.^15^ In conclusion, cerebral hemangiopericytomas are rare tumours of the brain, and they can present with atypical symptoms such as the one in our case with contralateral epilepsia partialis continua. Although this type of clinical picture has not been reported in the literature before, it should be understood that due to the rarity of this disease, the various ways of its presentation have not yet been discovered. Imaging can be confusing with meningioma, and the diagnosis can only be confirmed by immunohistochemistry. Hemangiopericytomas are highly vascular and thus carry a risk of massive intraoperative bleeding. They frequently recur and can metastasize axially and extra axially so the patients should be closely monitored on follow up.
